# Antioxidant vitamins promote anticancer effects on low‐concentration methotrexate‐treated glioblastoma cells via enhancing the caspase‐3 death pathway

**DOI:** 10.1002/fsn3.2298

**Published:** 2021-05-01

**Authors:** Giou‐Teng Yiang, Tsu‐Yi Chen, Cian Chen, Yu‐Ting Hung, Kuan‐Chun Hsueh, Tsai‐Kun Wu, Ying‐Ru Pan, Yi‐Chung Chien, Chao‐Hsuan Chen, Yung‑Lung Yu, Chyou‐Wei Wei

**Affiliations:** ^1^ Department of Emergency Medicine Taipei Tzu Chi Hospital Buddhist Tzu Chi Medical Foundation New Taipei Taiwan; ^2^ Department of Emergency Medicine School of Medicine Tzu Chi University Hualien Taiwan; ^3^ Master Program of Biomedical Nutrition Department of Nutrition Hung kuang University Taichung Taiwan; ^4^ Graduate Institute of Biomedical Sciences China Medical University Taichung Taiwan; ^5^ Department of Surgery Tungs' Taichung MetroHarbor Hospital Taichung Taiwan; ^6^ Division of Renal Medicine Tungs' Taichung MetroHarbor Hospital Taichung Taiwan; ^7^ Drug Development Center Research Center for Cancer Biology China Medical University Taichung Taiwan; ^8^ Center for Molecular Medicine China Medical University Hospital Taichung Taiwan; ^9^ Department of Neurosurgery China Medical University Hospital Taichung Taiwan; ^10^ Ph.D. Program for Translational Medicine China Medical University Taichung Taiwan; ^11^ Institute of New Drug Development China Medical University Taichung Taiwan; ^12^ Department of Medical Laboratory Science and Biotechnology Asia University Taichung Taiwan

**Keywords:** antioxidant, glioblastoma, methotrexate, vitamin C, vitamin E

## Abstract

Vitamin C and vitamin E are well‐known antioxidant vitamins, both of which are also applied as adjunct treatments for cancer therapy. Methotrexate (MTX) is a clinical drug that is used widely for rheumatoid arthritis and cancer treatment. Human glioblastoma multiforme (GBM) is an aggressive malignant brain tumor; the mean survival time for GBM patients is <2 years with traditional therapies. Developing and investigating novel treatments are important for clinical GBM therapy. Therefore, the aim of this study was to investigate whether combined treatment with vitamin C/E and MTX can display anticancer activities on GBM. Our studies showed that MTX displays anticancer effects on GBM in a dose‐dependent manner, while vitamins C and E are not cytotoxic to glioblastoma. Importantly, this study showed that vitamins C and E can promote anticancer effects on low‐concentration methotrexate‐treated glioblastoma. Additionally, this study suggested that MTX alone or combined with vitamins C/E inhibits GBM cell growth via the caspase‐3 death pathway.

## Graphical abstract

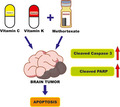

## 1 INTRODUCTION

Human glioblastoma multiforme (GBM) is a malignant, and often lethal, central nervous system tumor (Chen et al., 2015; Gokturk et al., 2018). Current clinical treatments include surgery, chemotherapy, and radiotherapy (Daneyemez et al., 1998; Yiang et al., 2013). Temozolomide is a commonly used chemical drug in GBM treatment (Gokturk et al., 2018); however, GBM is a high‐grade malignant brain cancer and has a poor prognosis. The survival time of most patients is about 1–3 years (Baillie et al., 2018; Daneyemez et al., 1998; Tovilovic‐Kovacevic et al., 2018; Yiang et al., 2013). Potential therapies, required for GBM treatment, are currently being studied (Hegde et al., 2014; Qin et al., 2014).

Vitamins C and E are common antioxidant nutrients (Lee et al., 2018; Panebianco et al., 2019), and both have been used in cancer treatment (Banks et al., 2010; Chang & Yu, 2016; Jiang, 2019; Lee, 2009; Nagappan et al., 2012). Many reports have shown that vitamin C can enhance conventional anticancer drug‐induced cytotoxicity on cancer cells (Guerriero et al., 2014; Vetvicka & Vetvickova, 2012; Wu et al., 2017). Additionally, previous studies also indicated that vitamin E can lower cancer risk and inhibit cancer growth (Chamras et al., 2005; Constantinou et al., 2008; Smolarek & Suh, 2011). Some studies have also reported that vitamin C is applied as an adjuvant drug for GBM therapy in order to improve anticancer effects and decrease cancer therapy‐related side effects (Baillie et al., 2018; Rodríguez et al., 2013). Previous studies also showed that vitamin E can decrease GBM risk and improve the quality of life for GBM patients (Di Bella et al., 2015; Schwartzbaum & Cornwell, 2000). A recent study suggested that the question of whether vitamin E is beneficial as a supportive therapy for GBM patients merits further investigation (Mulpur et al., 2015).

Methotrexate (MTX), a folate antagonist, is usually used for rheumatoid arthritis treatment (Whittle & Hughes, 2004; Wu et al., 2017) but is also used in various cancer treatments (Plas et al., 2015; Wei et al., 2019). Many studies indicated that MTX displays anticancer effects on various cancers such as breast cancer, hepatoma, leukemia, lymphoma, and gastric cancer cells (Plas et al., 2015; Shirao et al., 2013; Wei et al., 2019; Yiang et al., 2014). MTX has not been extensively studied as a GBM treatment because, like many drugs, it is inefficient at crossing the blood–brain barrier (BBB) (Capeloa et al., 2014). Although MTX‐induced anticancer effects remain to be studied, MTX has been applied in some GBM treatments; a clinical report used low‐dose combination treatment with methotrexate and cyclophosphamide for recurrent glioblastoma treatment (Herrlinger et al., 2005). In addition, combination hyperosmotic disruption of the BBB with MTX has been used for GBM treatment (Morikawa et al., 1999, 2000). Currently, novel methods are being developed to increase MTX permeability to cross the BBB for GBM treatment (Capeloa et al., 2014; Pereira et al., 2018; Ye et al., 2018). These studies indicated that MTX may be a potential drug for GBM therapy, but high‐dose MTX can induce oxidative stress and cause serious side effects (Singh et al., 2015; Turkler et al., 2020). A previous clinical report also suggested that high‐dose MTX treatment should be applied cautiously in GBM patients (Price et al., 2008). Therefore, it is valuable to investigate how to reduce MTX‐induced side effects and enhance low‐dose MTX‐induced anticancer effects. Today, combination treatments with antioxidant vitamins C/E with MTX are used for GBM treatment. Our study demonstrates that antioxidant vitamins can promote anticancer effects on low‐concentration methotrexate‐treated glioblastoma multiforme.

## 2 MATERIALS AND METHODS

### 2.1 Materials

Vitamins C and E were obtained from Sigma‐Aldrich (St. Louis). Anti‐tubulin (1:1,000; cat. no. BS1699) polyclonal antibody was bought from Bioworld (Louis Park). Anti‐cleaved PARP (1:2,000; cat. no. 9544) polyclonal antibody, anti‐caspase‐3 (1:1,000; cat. no. 9965) polyclonal antibody, and horseradish peroxidase (HRP)‐conjugated goat anti‐rabbit IgG secondary antibody (1:2,000, cat. no. 7074) were obtained from Cell Signaling Technology (Danvers). The MTT assay kit was obtained from BIO‐BASIC CANADA INC (Markham). Fetal bovine serum, Dulbecco's modified Eagles medium (DMEM), nonessential amino acids, L‐glutamine, and penicillin/streptomycin were obtained from GIBCO BRL (Invitrogen Life Technologies).

### 2.2 Cell lines and cell culture

The human glioblastoma DBTRG and human renal tubular epithelial (HK‐2) cells were bought from the Bioresource Collection and Research Center (Shin Chu). The cells were cultured and maintained in DMEM medium containing 10% fetal bovine serum, 2 mM L‐glutamine, 100 IU/ml penicillin/streptomycin, and 0.1 mM nonessential amino acids. The cells were maintained at 37°C in a humidified 5% CO_2_ atmosphere.

### 2.3 Cell viability assay

Cell viability was determined by using the MTT assay as described in previous studies (Wu et al., 2018; Yu et al., 2014). In brief, cells were cultured into 96‐well plates. Every 24 hr, the control and experimental cells were treated with the MTT kit. After 3 hr incubation at 37°C, the formazan products were dissolved and determined at 570 nm (A570) by using a Multiskan™ FC Microplate Photometer (Molecular Devices). The cell viability was calculated as (A570 experimental group)/(A570 control group) × 100%.

### 2.4 SDS electrophoresis and Western blot assay

Control and experimental cells were collected and lysed in radio‐immunoprecipitation assay (RIPA) buffer (cat. no. 20–188; EMD Millipore). After centrifugation (16,000 × *g*; 4°C) for 20 min, cellular protein was obtained from the supernatant layer. The protein concentration was determined with a protein assay kit (cat. no. 23200; Thermo Fischer Scientific, Inc.). Equal quantities (30 μg) of protein were separated in a 13.3% SDS gel and transferred onto polyvinylidene difluoride membranes (EMD Millipore). After washing in phosphate‐buffered saline (PBS), the membranes were blocked with 5% nonfat milk for 2 hr at room temperature. After washing with PBS, the membranes were treated with primary antibodies for 4 hr. Next, the membranes were washed with PBS and treated with anti‐rabbit HRP‐conjugated secondary antibodies for 1 hr at room temperature. The immunolabeled proteins were treated with Western Lightning^®^ Chemiluminescence Plus reagent (PerkinElmer, Inc.) and determined with a Luminescence Image Analysis system (LAS‐4000, FUJIFILM Electronic Materials Taiwan Co., Ltd.).

### 2.5 Statistical analysis

All data were collected from four independent experiments. The data were calculated as the mean ± *SEM*. Values were analyzed using ANOVA post hoc test (SPSS for Windows, version 10; SPSS, Inc.). The *p*‐value <0.05 was considered statistically significant.

## 3 RESULTS

### 3.1 Methotrexate inhibits DBTRG cell growth in a concentration‐dependent manner

**FIGURE 1:**
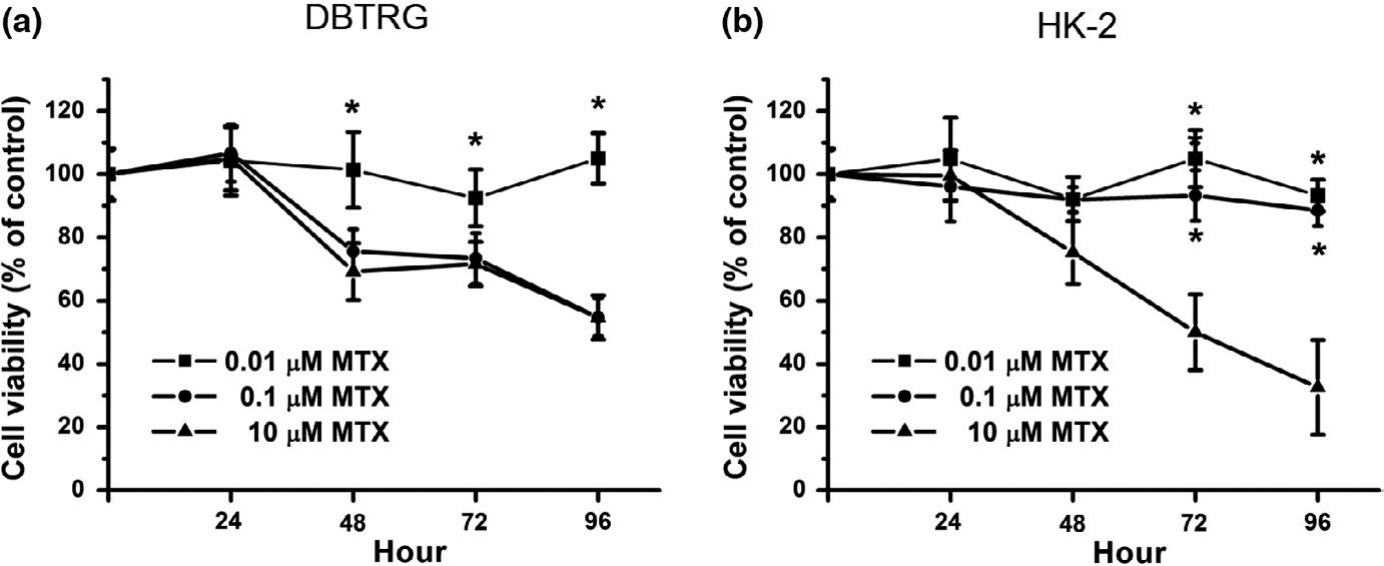
Cell viability on MTX‐treated DBTRG and HK‐2 cells. (a) DBTRG cells were treated with 0.01, 0.1, or 10 μM MTX for 96 hr. (b) HK‐2 cells were treated with 0.01, 0.1, or 10 μM MTX for 96 hr. Cell viability was determined by MTT assay and calculated as A570 experimental group/A570 control group × 100%. Data were from four independent experiments and presented as mean ± *SD*. The * represents *p* < .05, compared with 10 μM MTX‐treated group

The cytotoxicity effects of various concentrations of MTX on human glioblastoma multiforme (DBTRG cells) were examined. In this study, the cell viability was above 95% for 0–96 hr for DBTRG cells with low‐dose MTX (0.01 µM) treatment (Figure 1a). The cell viability gradually decreased over 0–96 hr for DBTRG cells with 0.1 and 10 µM MTX treatments (Figure 1a). The cell viability was about 50% on MTX (0.1 and 10 µM)‐treated cells at 96 hr. Our study indicated that MTX can inhibit DBTRG cell growth in a concentration‐dependent manner. The cytotoxicity effects of various concentrations of MTX on human renal tubular epithelial cells (HK‐2) were also examined (Figure 1b). The data showed that only 10 µM MTX is cytotoxic to HK‐2 cells, while 0.1 and 0.01 µM MTX are not cytotoxic to HK‐2 cells. Our study indicated that although 10 µM MTX displayed anticancer activity on DRTRG cancer cells, 10 µM MTX also induced cytotoxicity on normal HK‐2 cells.

### 3.2 Antioxidant vitamins are not cytotoxic to DBTRG and HK‐2 cells

**FIGURE 2:**
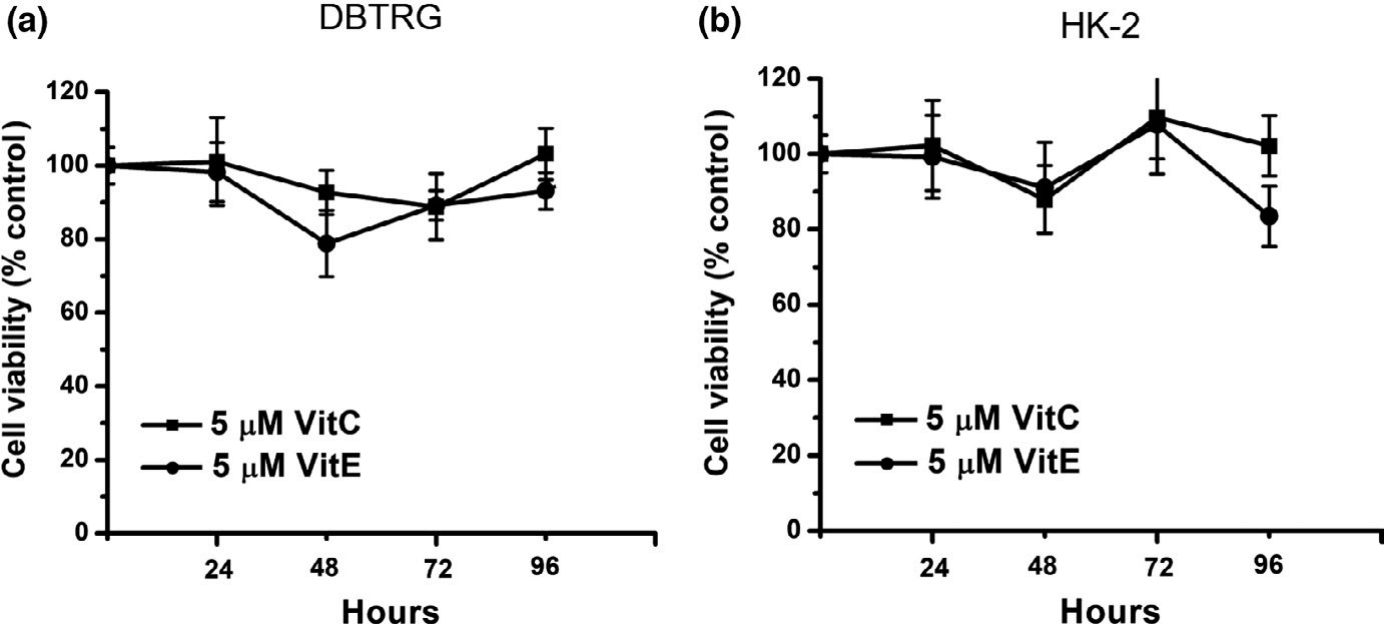
Cell viability on vitamin‐treated DBTRG and HK‐2 cells. (a) DBTRG cells were treated with 5 μM vitamin C or vitamin E for 96 hr. (b) HK‐2 cells were treated with 5 μM vitamin C or vitamin E for 96 hr. Cell viability was determined by MTT assay and calculated as A570 experimental group/A570 control group × 100%. Data were from four independent experiments and presented as mean ± *SD*

Many studies have shown the effects of using antioxidant vitamins in clinical cancer therapy. Therefore, we examined the cytotoxic effect of vitamin C and vitamin E on DBTRG cells. Our data showed that both vitamin C (5 µM) and vitamin E (5 µM) are not cytotoxic to DBTRG cells (Figure 2a). This study also examined the cytotoxic effect of vitamin C and vitamin E on HK‐2 cells. Our data also showed that both vitamin C (5 µM) and vitamin E (5 µM) are not cytotoxic to HK‐2 cells (Figure 2a).

### 3.3 Vitamin C decreases cell viability in low‐concentration MTX‐treated cells

**FIGURE 3:**
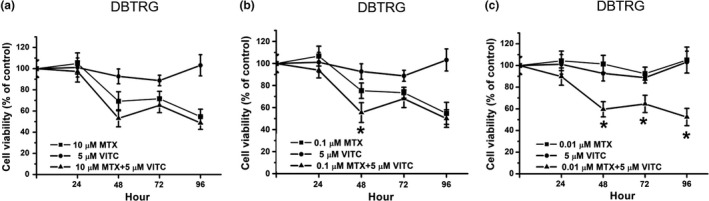
Cell viability on MTX, vitamin C and MTX plus vitamin C‐treated DBTRG cells. (a) DBTRG cells were treated with 10 μM MTX, 5 μM vitamin C or 10 μM MTX plus 5 μM vitamin C. (b) DBTRG cells were treated with 0.1 μM MTX, 5 μM vitamin C or 0.1 μM MTX plus 5 μM vitamin C. (c) DBTRG cells were treated with 0.01 μM MTX, 5 μM vitamin C, or 0.01 μM MTX plus 5 μM vitamin C. Cell viability was determined by MTT assay and calculated as A570 experimental group/A570 control group × 100%. Data were from four independent experiments and presented as mean ± *SD*. The * represents *p* < .05, compared with MTX‐treated group

Next, the effect of combined treatment with 5 µM vitamin C and various concentrations of MTX was determined. Compared with 10 µM MTX‐treated cells, the 10 µM MTX plus vitamin C‐treated cells had a similar cell viability (Figure 3a). Compared with 0.1 µM MTX‐treated cells, the 0.1 µM MTX plus vitamin C‐treated cells also had a similar cell viability (Figure 3b). That is, vitamin C does not influence cytotoxic effects in 10 and 0.1 µM MTX‐treated cells. Compared with 0.01 µM MTX‐treated cells, the 0.01 µM MTX plus vitamin C‐treated cells had a significantly lower cell viability from 48 to 96 hr (Figure 3c). Our results suggest that vitamin C can decrease cell viability in low‐concentration MTX‐treated cells. That is, vitamin C can enhance cytotoxic effects in low‐concentration MTX‐treated DBTRG cells.

### 3.4 Vitamin E decreases cell viability in low‐concentration MTX‐treated cells

**FIGURE 4:**
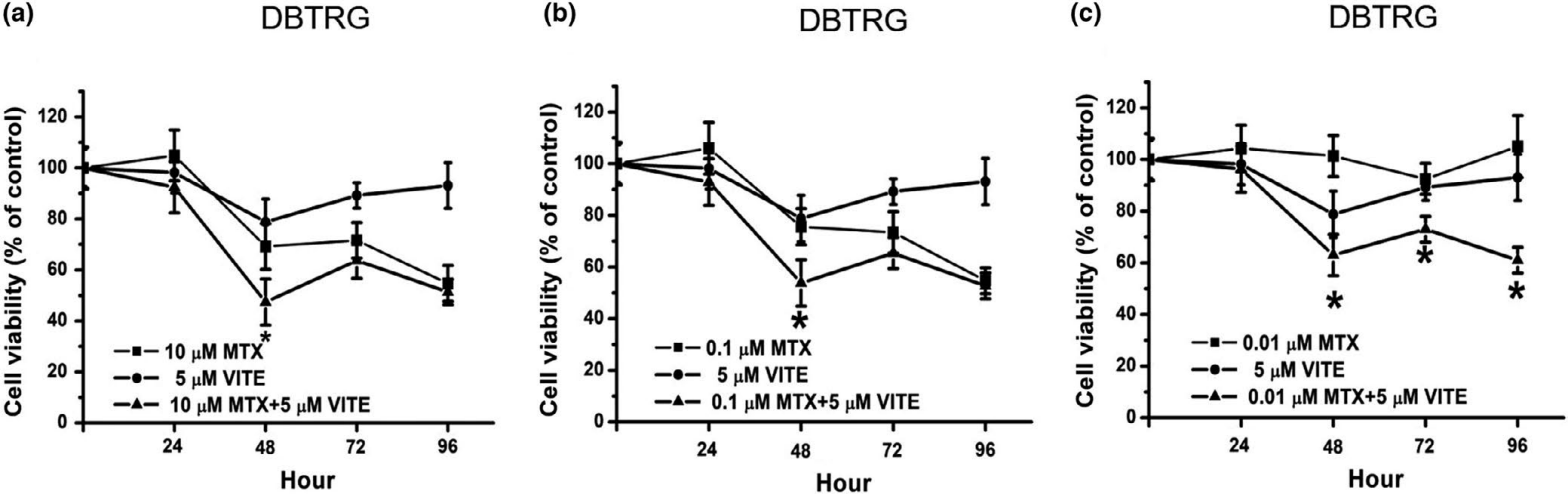
Cell viability on MTX, vitamin E, and MTX plus vitamin E‐treated DBTRG cells. (a) DBTRG cells were treated with 10 μM MTX, 5 μM vitamin E, or 10 μM MTX plus 5 μM vitamin E. (b) DBTRG cells were treated with 0.1 μM MTX, 5 μM vitamin E or 0.1 μM MTX plus 5 μM vitamin E. (c) DBTRG cells were treated with 0.01 μM MTX, 5 μM vitamin E, or 0.01 μM MTX plus 5 μM vitamin E. Cell viability was determined by MTT assay and calculated as A570 experimental group/A570 control group × 100%. Data were from four independent experiments and presented as mean ± *SD*. The * represents *p* < .05, compared with MTX‐treated group

Another antioxidant, vitamin E (5 µM), was also examined in this study. Compared with the 10 µM MTX‐treated group, the 10 µM MTX plus vitamin E‐treated group did not show enhanced cytotoxic effects on DBTRG cells (Figure 4a). Compared with the 0.1µM MTX‐treated group, the 0.1 µM MTX plus vitamin E‐treated group also did not show increased cytotoxic effects on DBTRG cells (Figure 4b). The data indicated that vitamin E does not influence cytotoxic effects in 10 and 0.1 µM MTX‐treated DBTRG cells. Compared with the 0.01 µM MTX‐treated group, the 0.01 µM MTX plus vitamin E‐treated group displayed a significantly lower cell viability from 48 to 96 hr (Figure 4c). Our data indicated that vitamin E can promote cytotoxic effects in low‐concentration MTX‐treated DBTGR cells.

### 3.5 The cytotoxic effects on HK‐2 cells with vitamins C/E plus MTX treatments

**FIGURE 5:**
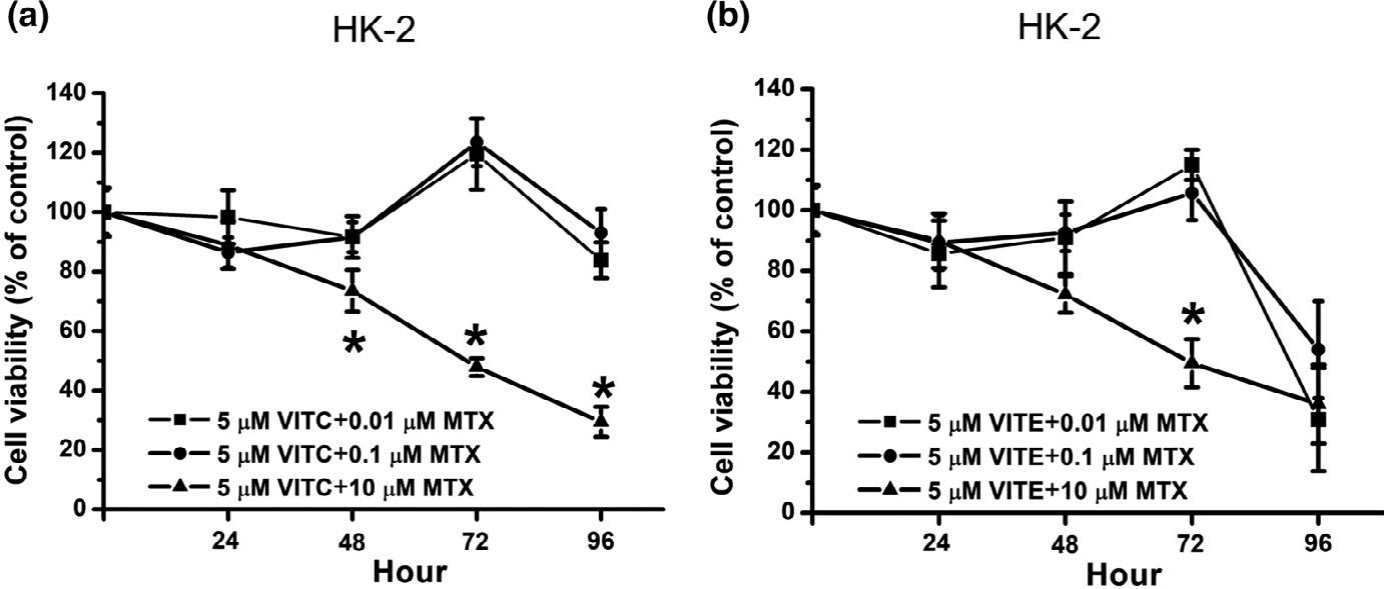
Cell viability on MTX plus vitamin C or vitamin E‐treated HK‐2 cells. (a) HK‐2 cells were treated with various concentration MTX (10, 0.1, and 0.01 μM) plus 5 μM vitamin C. (b) HK‐2 cells were treated with various concentration MTX (10, 0.1, and 0.01 μM) plus 5 μM vitamin E. Cell viability was determined by MTT assay and calculated as A570 experimental group/A570 control group × 100%. Data were from four independent experiments and presented as mean ± *SD*. The * represents *p* < .05, compared with 0.01 μM MTX plus vitamin‐treated group

This study further determined the cytotoxic effects on normal HK‐2 cells with vitamins C/E plus MTX treatments. As shown in Figure 5a, vitamin C plus 0.1 or 0.01 µM MTX treatments did not induce cytotoxicity in HK‐2 cells, while vitamin C plus 10 µM MTX decreased cell viability in HK‐2 cells after 24–96 hr. As shown in Figure 5b, vitamin E plus 0.1 or 0.01 µM MTX was not cytotoxic to HK‐2 cells from 0 to 72 hr, however, the cell viability was decreased at 96 hr. In addition, cell viability decreased in HK‐2 cells with vitamin E plus 10 µM MTX treatment from 24 to 96 hr. Taken together, our data suggest that vitamin C plus 0.1 or 0.01 µM MTX is safer than other treatments in normal HK‐2 cells.

### 3.6 PARP cleavage and caspase‐3 activation are induced in MTX‐treated and MTX plus vitamin‐treated cells

**FIGURE 6:**
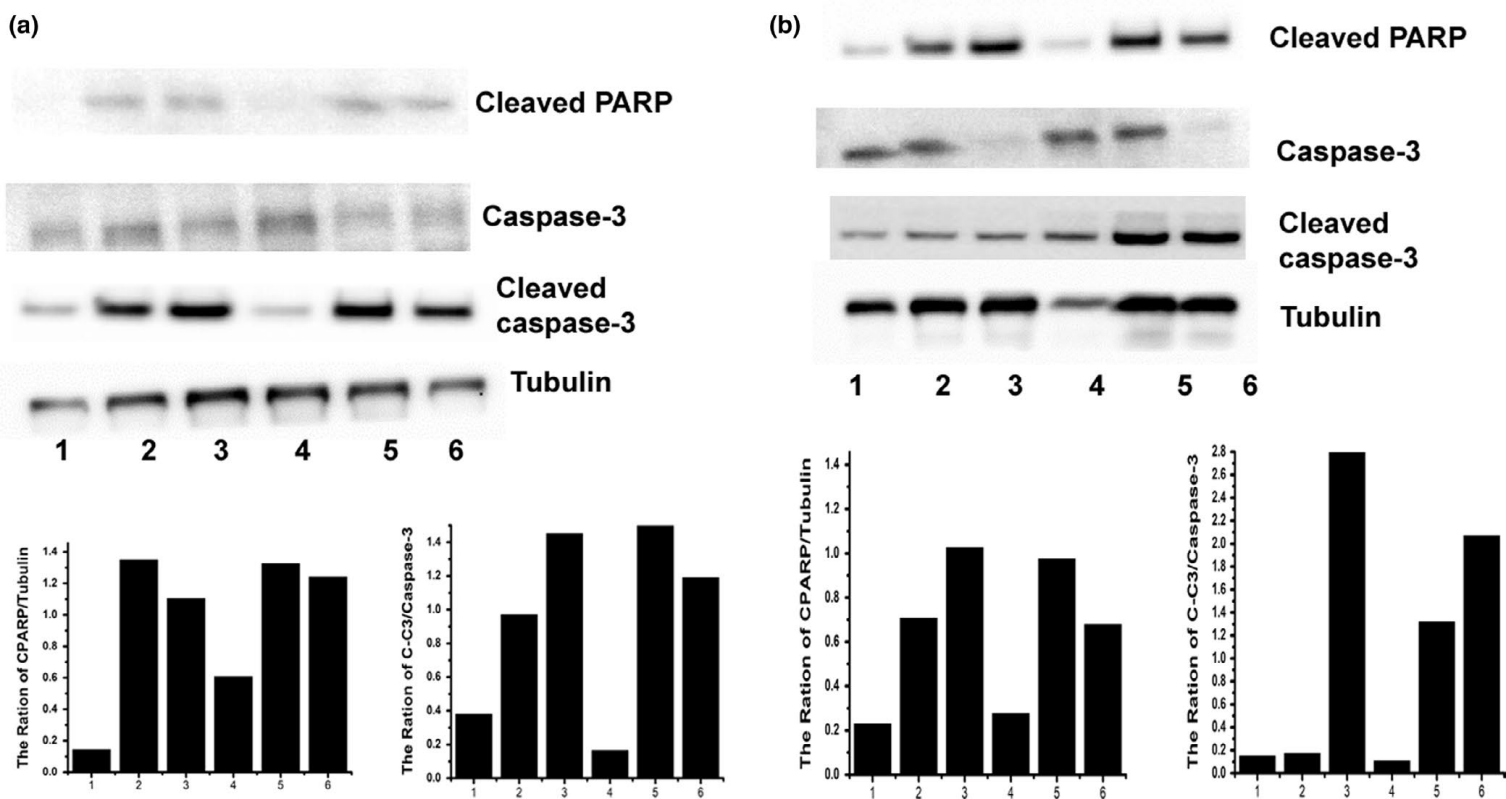
PARP cleavage and caspase‐3 activation were observed by Western blotting. (a) DBTGR cells were treated with MTX, vitamin C and MTX plus vitamin C. (b) DBTGR cells were treated with MTX, vitamin E, and MTX plus vitamin E. Lane 1: control; lane 2:10 μM MTX; lane 3:0.1 μM MTX; lane 4:5 μM vitamin; lane 5:10 μM MTX plus 5 μM vitamin; lane 6:0.1 μM MTX plus 5 μM vitamin. Tubulin was used as a loading control

This study further investigated whether the caspase‐dependent death pathway is involved in MTX‐induced and MTX plus vitamin‐induced cytotoxicity in DBTRG cells. PARP is a substrate of caspase‐3; therefore, cleaved PARP was found when caspase‐3 was activated. Two forms of caspase‐3 may exist in cells, including procaspase‐3 and cleaved caspase‐3. Cleaved caspase‐3 is the activated form of caspase‐3. Today, cleaved PARP and caspase‐3 activation were examined by Western blotting and the ration of cleaved PARP (CPARP)/Tubulin as well as the ration of cleaved caspase‐3 (C‐C3)/caspase‐3 were indicated in the Figure 6. As shown in Figure 6a, compared with the control group, the cleaved PARP level was higher in MTX‐treated and MTX plus vitamin C‐treated groups. In addition, compared with the control group, the cleaved caspase‐3 level was also higher in MTX‐treated and MTX plus vitamin C‐treated groups. Our data indicated that both MTX alone and MTX plus vitamin C induce cytotoxicity in DBTRG cells; that is related to the caspase‐dependent death pathway. Similar results were found in DBTRG cells treated with MTX plus vitamin E. As shown in Figure 6b, compared with the control group, cleaved PARP and cleaved caspase‐3 levels were higher in MTX‐treated and MTX plus vitamin E‐treated groups. That is, the caspase‐dependent death pathway is induced in MTX‐treated and MTX plus vitamin E‐treated cells. Taken together, our studies demonstrated that MTX alone, MTX plus vitamin C and MTX plus vitamin E can induce cytotoxicity in DBTRG cells through the caspase‐dependent pathway.

## 4 DISCUSSION

In this study, we determined the cell viability of DBTRG and HK‐2 cells with MTX alone treatment. As shown in Figure 1a, both 10 and 0.1 µM MTX could decrease cell viability in DBTRG cells. Our data show that only 10 µM MTX reduces the cell viability on HK‐2 cells while 0.1 and 0.01 µM MTX are not significantly cytotoxic to HK‐2 cells (Figure 1b). That is, 10 µM MTX has anticancer activity on DBTRG cells; however, 10 µM MTX also induces cytotoxic effects on HK‐2 cells. Compared with the 10 µM MTX treatment, the 0.1 µM MTX treatment displayed similar anticancer effects on DBTRG cells, while the 0.1 µM MTX treatment was not cytotoxic to HK‐2 cells. Therefore, our results suggest that 0.1 µM MTX is a better choice for DBTRG treatment than 10 µM MTX.

Vitamin C has antioxidant activities. Many studies have demonstrated that vitamin C can decrease oxidative stress against chemical therapy‐induced side effects for various cancer treatments (Jafari et al., 2018; Klimant et al., 2018; Vollbracht et al., 2011). Previous studies have also used vitamin C as an adjuvant drug for GBM treatment to decrease cancer therapy‐related side effects (Baillie et al., 2018; Rodríguez et al., 2013). Our study demonstrated that vitamin C can promote 0.01 µM MTX‐induced cytotoxic effects on DBTRG cells (Figure 3c). Compared with MTX treatment alone, our results showed that combination treatment with vitamin C and 0.01 µM MTX can express similar cell viability (Figure 3c) to 10 or 0.1 µM MTX alone (Figure 1a). Previous studies demonstrate low‐dose vitamin C with can reduce drug‐induced oxidative side effects while high‐dose vitamin C have anticancer activity. Today, our study suggested combination treatment with low‐dose vitamin C and MTX have anticancer activity. In addition, our study demonstrated that combination treatment with vitamin C and 0.01 µM MTX does not decrease cell viability in HK‐2 cells (Figure 5a). These results suggest that low‐concentration MTX plus antioxidant vitamin C treatment is a potential method for GBM therapy.

Vitamin E is an antioxidant nutrient. Previous studies suggest that vitamin E can decrease cancer therapy‐induced side effects (Gevrek & Erdemir, 2018; Magnusson et al., 2009). In addition, some studies indicated that vitamin E can reduce GBM risk and improve quality of life for GBM patients (Di Bella et al., 2015; Schwartzbaum & Cornwell, 2000); however, a recent study suggested that GBM patients using vitamin E as an adjuvant therapy merits further study (Mulpur et al., 2015). Our study showed that vitamin E can promote 0.01 µM MTX‐induced anticancer activities in DBTRG cells in 48–96 hr (Figure 4c). The data indicated that combination treatment with vitamin E and 0.01 µM MTX seems useful for GBM treatment. On the other hand, our study also showed that the cell viability is not decreased on vitamin E plus 0.01 µM MTX‐treated HK‐2 cells after 0–72 hr, while the cell viability decreased on vitamin E plus 0.01 µM MTX‐treated HK‐2 cells after 96 hr (Figure 5b). Therefore, our study considered whether using vitamin E as an adjuvant agent for GBM treatment requires further investigation.

A previous study demonstrated that 10 and 0.1 µM MTX alone is not cytotoxic to triple‐negative breast cancer cells (TNBC) (Wu et al., 2017). Compared with that study, our study showed that 10 and 0.1 µM MTX alone can induce anticancer effects on DBTRG cells. GBM may be more sensitive to MTX alone than TNBC. In addition, the previous study demonstrated that vitamin C enhanced 10/0.1 µM MTX‐induced anticancer effects on TBNC (Wu et al., 2017); however, our study showed that vitamin C can only enhance 0.1 µM MTX‐induced anticancer effects but vitamin C does not promote 10 µM MTX‐induced anticancer effects in DBTRG cells. The previous study and the current study suggest that combination treatment with vitamin C and higher‐dose MTX (10 and 0.1 µM) is required for TBNC treatment; however, combination treatment with vitamin C and lower‐dose MTX (0.01 µM) may be a better choice for GBM treatment. On the other hand, previous studies showed that combination treatment with vitamin C and MTX induces anticancer effects via the caspase‐3 signal pathway in TBNC and hepatoma (Wu et al., 2017; Yiang et al., 2014). Our study also demonstrated that combination treatment with vitamin C and MTX induces caspase‐3 activation in DBTRG cells.

Taken together, this study demonstrated that the antioxidants vitamins C and E can effectively promote anticancer activities in low‐concentration MTX (0.01 µM)‐treated DBTRG cells. In addition, combination treatment with vitamin C and MTX may be a potential method for GBM treatment; however, whether combination treatment with vitamin E and MTX is a beneficial therapy for GBM merits further study.

## CONFLICT OF INTEREST

The authors declare no conflict of interest.

## AUTHOR CONTRIBUTIONS

**Giou‐Teng Yiang:** Data curation (equal); Formal analysis (equal); Validation (equal); Writing‐original draft (equal). **Tsu‐Yi Chen:** Data curation (equal); Methodology (equal). **Cian Chen:** Formal analysis (equal); Methodology (equal). **Yu‐Ting Hung:** Methodology (equal); Resources (equal). **Kuan‐Chun Hsueh:** Methodology (equal); Resources (equal). **Tsai‐Kun Wu:** Methodology (equal); Resources (equal). **Ying‐Ru Pan:** Methodology (equal); Resources (equal). **Yi‐Chung** **Chien:** Methodology (supporting); Resources (supporting). **Chao‐Hsuan Chen:** Methodology (supporting); Resources (supporting). **Yung‐Luen Yu:** Project administration (equal); Supervision (equal); Writing‐review & editing (equal). **Chyou‐Wei Wei:** Project administration (equal); Supervision (equal); Writing‐review & editing (equal).

## Data Availability

Data and materials are available from the authors.
